# Fibroma of the Abdominal Wall

**Published:** 1906-12

**Authors:** J. Lacy Firth

**Affiliations:** Surgeon to the Bristol General Hospital


### FIBROMA OF THE ABDOMINAL WALL.

BY

J. Lacy Firth, M.S. Lond., F.R.C.S.,

*Surgeon to the Bristol General Hospital.*

---

Though the records of a large number of cases of fibroma of the abdominal wall have now been published—for example, C. Pfeiffer recently based a paper upon 400 cases—the disease is nevertheless uncommon. During a hospital experience of eighteen years it has only been my fortune to meet with one typical example, in which the diagnosis was verified by microscopical examination of the tumour after its removal, and this occurred recently. Some years previously I made a diagnosis of fibroma of the abdominal wall in the case of a woman who attended the Hospital out-patient department; but she ceased to attend after one or two visits, and the correctness of the diagnosis could not be tested. The woman had a hard mass in the abdominal parietes in front on the right side in the lumbar and hypochondriac regions.

The following are the clinical notes of the case I now wish to bring forward as a typical example of the affection :—

F. L., housewife, aged 29, was admitted to the Bristol General Hospital in January, 1905, with the following history.

She has had three children, and it is two and a half years since her last confinement. For two years she has been liable to attacks of pain in the left iliac and hypogastric regions of the abdomen and in the lower part of the back. The pain usually lasts two or three hours, but sometimes it persists for half a day. Rest relieves it, especially if she lies down. The pain in the back is more severe than that in front. During the first year the attacks of pain were at intervals of two or three months, but for the last year she has had them regularly every month without exception. They occur one week after menstruation. Four months ago, when lifting a tub of ten or twelve pounds weight, she was suddenly seized with a severe attack of the pain and had to keep her bed for two days, and the pain persisted for a week. It was on this occasion she made the discovery that she had a swelling in the left iliac region. She feels sure that no swelling existed there before, and that now, four months later, it is smaller than at first. She believes that the act of raising the tub produced the swelling. Since then the attacks of pain have occurred, as before, at monthly intervals, but they have been more severe.

*State.*—The patient is well nourished and apparently in good general health. There is an obvious slight prominence in the left iliac region, and palpation shows that this is due to an extremely hard, well-defined swelling of ovoid shape, with its long axis parallel to Poupart's ligament. The lower border of the mass is close to the ligament mentioned, the inner border reaches a point an inch and a half from the middle line, the outer border is an inch to the inner side of the anterior superior iliac spine, and the upper border is about three inches above the lower border. Contraction of the abdominal muscles obscures the borders of the swelling, but not the centre. When the muscles are flaccid the mass can be moved a little backwards and forwards and from side to side, but not from above downwards; also the fingers can be pressed beneath its upper and inner borders, and these borders can be raised. The swelling is not tender. A vaginal examination showed the absence of any pelvic swelling and of pelvic connections with the swelling in the iliac region.

A diagnosis of fibroma of the abdominal wall was made, and operation decided upon.

*Operation.*—An incision parallel with and about two inches above Poupart's ligament was made over the most prominent part of the swelling. The deeper layer of the superficial fascia was well defined and strong. The external oblique muscle and aponeurosis were much thinned, but completely covered the tumour. They were incised in the same line as the skin, and the flaps deflected upwards and downwards. This exposed the surface of the encapsuled tumour. Its upper border was intimately blended with the arched fibres of the internal oblique and transversalis muscles, and a narrow band of the same muscular tissue separated the lower border of the tumour from Poupart's ligament. These muscles were separated from the tumour, and the latter shelled out from the sub-peritoneal fat upon which it lay. The fat was abundant. There was an intimate connection between the inner end of the tumour and the anterior layer of the sheath of the rectus muscle. This was separated with the scalpel. After removal of the tumour a large gap was left between the fibres of the internal oblique which had been above the tumour and those which had been below. These two sets of fibres were joined with interrupted catgut sutures, which also passed through Poupart's ligament. The tension on these sutures was great, tending to split the muscles. The external oblique flaps were sutured, and also the deeper layer of the superficial fascia. Lastly, the skin wound was closed with Michell's sutures. The external abdominal ring was not exposed, nor was anything seen of the round ligament during the operation.

The wound healed by first intention, and twelve months later there was no sign of a recurrence of the tumour nor of weakness of the abdominal parietes at the seat of operation.

The tumour was hard and fibrous in appearance, and creaked under the knife when divided. It was roughly oviform. Macroscopically the section surface showed interlacing whitish fibres with darker areas of dense tissue between them. The mass was encapsuled. After soaking in formalin solution the capsule, together with a portion of the tumour substance proper, was peeled off. The tumour measured about five inches in the long axis and three inches in breadth and thickness. Microscopically it was found to consist of interlacing tracts of fibro-cellular connective tissue.

Fibromata of the abdominal wall, or desmoids as the Germans name them, are not tumours frequently met with, as I have stated above. Leon Labbé observed only ten cases in the Paris hospitals in the course of twenty years. Sanger collected seventy cases of tumour of the abdominal wall, of which sixty were fibromata. Ledderhose collected 100 cases in 1890. C. Pfeiffer's paper on the subject[1] is based on a consideration of 400 cases. These include 40 cases observed in Von Brun's clinic in the course of forty-six years, the 100 cases of Ledderhose, and 260 cases collected from other sources.

The tumours are met with in women much more frequently than in men, according to Pfeiffer in the proportion of 7 to 1. Women are affected most frequently in that period of life during which they most often bear children, between the ages of 25 and 35. In men the age-period in which the tumours are most frequently found is from 35 to 50.

The growths originate in the muscular and aponeurotic structures of the abdominal wall—hence the name desmoid which is given them—and most frequently in the lower part of the abdomen. According to Pfeiffer's statistics, 43 per cent. arise in the rectus muscle or its sheath, 17.5 per cent. in other abdominal muscles, and 17 per cent. in the superficial or deep abdominal fasciæ. Several muscles may be involved and the

[1] *Beitr. z. klin. Chir.*, 1904, xliv. 334; abstract in the *Zentralbl. f. Chir.*, 1905, xxxii. 114.

point of origin be undiscoverable. The structure is usually fibrous, more or less richly cellular. Rapidly-growing parts may resemble sarcoma in structure. Fibro-myomata are rare. Ætiologically the facts that these tumours in 89.4 per cent. of the cases affect women, and that of those affected women 94 per cent. have given birth to children, seem most significant. Distension of the abdominal wall apart from pregnancy is not a cause. In pregnancy there is hypertrophy of the abdominal wall; in pathological distension there is atrophy. Pfeiffer is inclined, with Ribbert, to attribute the tumours to the hypertrophic tendency induced by pregnancy persisting in circumscribed tracts of muscular or fascial tissue. Any cause of cell proliferation might similarly be the starting-point of such a tumour. The symptoms may be slight, and the tumour is often discovered accidentally by the patient. They may be discovered only after they have reached a large size, especially if the enlargement has been towards the abdominal cavity instead of outwards. They may grow slowly and steadily, or stop growing for a time and later begin to grow rapidly. In some cases they seem to become sarcomatous. If the tumour is near a bone the tendons and muscular fibres passing to it may be felt as a tense and firm band, simulating a bony pedicle.

The treatment should be early and complete removal. The peritoneum may be involved when the tumours are large and have to be resected more or less extensively, increasing the danger of the operation and the tendency to ventral hernia subsequently. In Pfeiffer's cases permanent cure followed operation in 50 per cent. of the men and in 90 per cent. of the women. Incomplete removal of the capsule may lead to early recurrence. Recurrence, when it takes place, is said by Senn to occur usually after a three-years' interval, so that a permanent cure cannot be assured before that period of time has elapsed.

